# Organoids and Microphysiological Systems: New Tools for Ophthalmic Drug Discovery

**DOI:** 10.3389/fphar.2020.00407

**Published:** 2020-04-03

**Authors:** Jing Bai, Chunming Wang

**Affiliations:** ^1^ Department of Mechanical Engineering, Massachusetts Institute of Technology, Cambridge, MA, United States; ^2^ State Key Laboratory of Quality Research in Chinese Medicine, Institute of Chinese Medical Sciences, University of Macau, Taipa, Macau

**Keywords:** organoid, microphysiological system, ocular, organ-on-a-chip, 3D tissue constructs

## Abstract

Organoids are adept at preserving the inherent complexity of a given cellular environment and when integrated with engineered micro-physiological systems (MPS) present distinct advantages for simulating a precisely controlled geometrical, physical, and biochemical micro-environment. This then allows for real-time monitoring of cell-cell interactions. As a result, the two aforementioned technologies hold significant promise and potential in studying ocular physiology and diseases by replicating specific eye tissue microstructures *in vitro*. This miniaturized review begins with defining the science behind organoids/MPS and subsequently introducing methods for generating organoids and engineering MPS. Furthermore, we will discuss the current state of organoids and MPS models in retina, cornea surrogates, and other ocular tissue, in regards to physiological/disease conditions. Finally, future prospective on organoid/MPS will be covered here. Organoids and MPS technologies closely recapture the *in vivo* microenvironment and thusly will continue to provide new understandings in organ functions and novel approaches to drug development.

## Introduction

Organoid and microphysiological system are two emerging techniques to recapitulate the key organ features. Most of the current conventional cell culture systems, for example, Transwells are considered as oversimplified *in vitro* models, and thus are not appropriate platforms to study organ functions. Organoids, which are miniaturized organs with a three-dimensional structure and multiple cell layers, have advantages over two-dimensional models by maintaining organ anatomic microstructure and basic organ functions (Lancaster and Knoblich, 2014). Organoids are either generated from primary tissue cells, embryonic stem cells, or induced pluripotent stem cells (iPSCs). They possess the capability of self-assembly, self-renewal, and differentiation, ranging in size from micrometer to millimeter scale, with the potential to address the limitations of conventional cell culture system, such as random configuration (Liu et al., 2019).

Microphysiological system (MPS), also known as organ-on-a-chip technology, is an integrative, microfabricated platform designed to recapitulate functional units of human organs *in vitro* (Huh et al., 2011). Conventional 3D models, where cells are growing within the extracellular matrix (ECM), fail to reflect critical aspects of human organs, including cell-tissue interface and physical and biochemical stimuli, such as flow and pressure. MPS provides essential advantages in this regard. First, fluid is restrained in micro-scale channels, enabling close contact between different cell types (e.g., between epithelium and vascular endothelium) to capture the dynamic cell-cell interplay. Next, microenvironment cues, including spatiotemporal gradients of chemicals and mechanical strain that are critical to mimic the organ functions, can be examined in a single microfluidic setup. In addition, vasculature-on-a-chip enables emulating pulsatile blood luminal flow and interstitial flow, the key determinants of tissue functions (Kim et al., 2017); and these vascular modules are embedded in interconnected multiple tissue units for blood supply. Lastly, in MPS, the optical transparency of microfluidic devices allows for real-time monitoring. Optical tracking of cell migration and cell-cell interaction presents an indispensable tool for analyzing tissue functions, such as monitoring the dynamic process of tissue development, repair, and regeneration (Tkachenko et al., 2009). **Table 1** compares organoids and MPS in their definition, functions, and applications. These two techniques are conceptually different, yet complimentary toward the same goal of recapitulation organ functions *in vitro* (Park et al., 2019).

**Table 1:** Comparison of organoids and microphysiological systems (MPS) on their definition, functions, and applications.

	Organoids 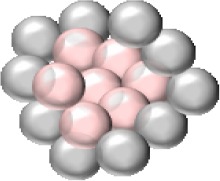	Microphysiological systems ** 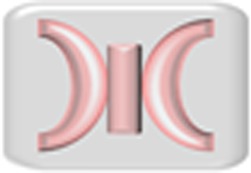 **
**Structure**	3D *in vitro,* • Self-organized functional organotypic units	3D *in vitro* • Microfabricated cell culture device recapitulating organ functional units
**Size**	μm–mm	μm–mm
**Easy-to-culture**	Moderate • Timely activation of cell-fate specification signaling pathways	Easy–moderate • Explicit structuring patterning—e.g., bioprinting, bio lithography
**Culture time**	Moderate • Weeks	Short–moderate • Days to weeks
**Co-culture ability**	Yes	Yes • Limited cell types in device
**Reproducibility**	Moderate • Lack of understanding and guide of their differentiation	Yes • Appropriate culture conditions and microfabrication
**High-throughput**	Low	Moderate–low
**Microenvironment**	Absent • Oversimplified models in absent of flow, physical and chemical cues	Present • Complex models replicating organ functions, e.g., muscle contractions, blood flow
**Vasculature and blood perfusion**	Highly possible	Highly possible
***In vivo*-like function**	Moderate • Recapitulating basic organ functions	High • Emulating complex organ functions
**Genetic manipulation**	Applicable • Inherited key genetic features from iPSCs	Applicable
**Application to personalized medicine and drug screening**	Yes	Yes

The technical advancement on microfluidics, 3D printing, tissue engineering materials, and microfabrication has paved the way for the development of novel MPS. This easy-to-use platform can be replicated in standard biological labs for development and tissue regeneration study, and enable clinical researchers to optimize drug treatment. In addition, there is growing recognition in industrial partners that this technique is suitable for pre-clinic drug screening and toxicology evaluation. The benefits of this 3D miniaturized assays include reducing animal test, evaluating targeted drug delivery and accelerating drug development process.

**Figure 1:**
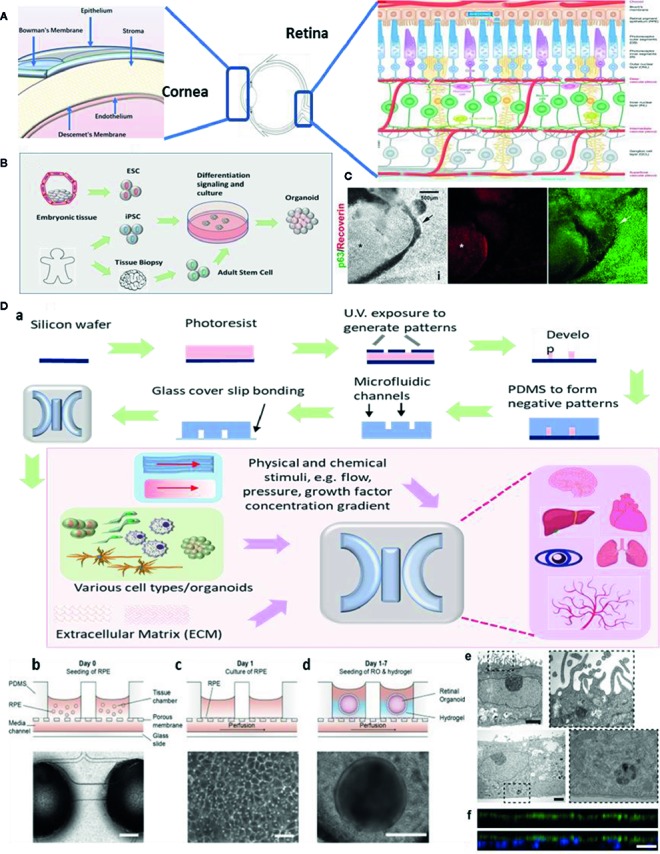
Ocular organoids and microphysiological systems (MPS). **(A)** Structure of eye, cornea, and retina. Retina structure was adapted from (Fu et al., 2019). **(B)** Schematic representation on the stepwise generation of organoids. **(C)** Differentiation of human iPSCs into retinal and corneal organoids. Distinct circular to oval-shaped eye field primordial (EFP) encompassed a centrally located p63^+^ Recoverin^+^ neuroretinal (NR) cup (asterisks), adapted from (Susaimanickam et al., 2017). **(D)** a. Schematic representation of photolithography for polydimethylsiloxane (PDMS)-based microfluidic device fabrication and essential components in a standard MPS model. b.c.d.e.f. An representative example of merging organoid and MPS technology in a human retina-on-a-chip platform: b.c.d. microfluidic platform enabling co-culture of human induced pluripotent stem cell (hiPSC)-derived retinal pigment epithelium (RPE) and ROs (retinal organoids) in a defined physiological structure (the top layer has compartments for the ROs and RPE, and the bottom layer with a channel for a vasculature); e.f. electron microscopic image of polarized RPE cells. RPE cells display apical microvilli (top row) and a basal membrane (bottom row), and immunohistochemical staining for ezrin (green), an apical microvilli marker, indicated polarized RPE, adapted from (Achberger et al., 2019).

Eye diseases like macular degeneration, glaucoma, and cataracts are not self-healing and can cause severe visual impairment. Common retinal disorders, including age-related macular degeneration (AMD), retinitis pigmentosa, and diabetic retinopathy (Zhang et al., 2015), affect millions of people world-wide. Meanwhile, ocular surface diseases remain another therapeutic challenge for vision restoration. It is crucial to investigate the pathogenesis of ocular diseases and to develop novel drugs. Considerable progress has been achieved on developing physiological and pathological ocular models, aiming to recapitulate key aspects in the ocular development and diseases. Despite that organoids and MPS models in major organs has been developed, such as liver (Wu et al., 2019), heart (Nugraha et al., 2019), brain (Qian et al., 2019), and pathological phenotypes like cancer (Niu et al., 2014; Tu et al., 2014; Bai et al., 2015a; Adriani et al., 2016) and Alzheimer’s disease (Gonzalez et al., 2018), remarkable achievement has been made to study ocular organoids and MPS. Although these techniques are still simplified methods, they have shown the potential to capture key features of basic ocular tissues like cornea, retina, and lens (**Figure 1A**). In this review, we will discuss the methods to generate organoids and MPS in ophthalmic research and focus on the most promising models, including retinal, corneal, and other ocular tissue models. We also acknowledge the future prospective of these novel technologies and benefits on drug discovery.

### Generating Organoids and Engineering Micro-Physiological Systems

Organoids are generated from either primary cells or progenitor cells isolated from biopsy, or pluripotent stem cells (PSCs) (**Figure 1B**). With sequential differentiation steps, the cells would be capable to retain the intrinsic developmental features of an organ. The most commonly used cells are iPSCs. These cells are PSCs that can be generated directly from adult cells with four reprogramming factors Oct3/4, Sox2, Klf4, and c-Myc (Takahashi and Yamanaka, 2006). The advantage of iPSCs is their high potential to differentiate into all major tissues, cell lineages, and high degree of biomimicry. A review has summarized organoid generation methods and discussed a promising approach of “human on a dish”(McCauley and Wells, 2017). Briefly, iPSCs were differentiated and self-assembled to generate organotypic constructs with extrinsic signals, including growth factors, extracellular matrix, and culture media. The 3D cellular structures could recapitulate the developmental features after 2 weeks and could be maintained in culture for months.

Technical advances in organoid fabrication provide hope for generating more complex MPS prototypes. MPS assembles the microphysical and chemical microenvironment. Successful fabrication of MPS requires two major techniques: microfluidics which precisely control a small amount of fluid to mimic interstitial flow in the organs; and microfabrication which assembles the essential 3D compartments and microchannel networks for organoids/cells to grow. Previous microfabrication method used silicon until soft lithography of polydimethylsiloxane (PDMS) replica became the most dominant method for organ-on-a-chip applications (**Figure 1D**). This is because of its easy fabrication, outstanding optical transparency and minimum cytotoxicity (Huh et al., 2011; Ghaemmaghami et al., 2012). A detailed review article has discussed PDMS for organ-on-a-chip applications (Sia and Whitesides, 2003). More recently, 3D bioprinting has been widely applied to the fabrication of organ microstructures. This technology has streamlined the fabrication process of microfluidic compartments to one single step (Ho et al., 2015). 3D bioprinting is capable of integrating multiple cells, matrix components, and growth factors, allowing for cells to assemble along more exact layer-by-layer printed microstructures. Typical 3D bioprinting methods involve micro-extrusion, inkjet, and laser-assisted printing (Yi et al., 2017), while micro-extrusion is the most common method for microfluidic chips fabrication. Another benefit of 3D bioprinting is that the printing ink can consist of any natural or synthetic biocompatible material (depending on the requirement of the design).

### Retina Models

The retina is the innermost layer of the eye with a complex light-sensing structure. A good review has described the anatomy and function of retina by Hoon et al. (2014) (**Figure 1A**, right). Approaches have emerged for developing retinal organoids and MPS. One of the most well-known ocular organoid models is the self-organizing optic-cup (Eiraku et al., 2011) differentiated from mouse embryonic stem cell.

Modeling retinal organoids and MPS is challenging because the retina has one of the most complex and fine structures in the body with a high variety of cell types. Therefore, only partial aspects of its functions are capable to be recapitulated in a single model. Thus far, efforts have been made to develop two perspectives of retinal structure. First, the neurosensory structure including photoreceptor cells, ganglion cells, and optic nerve; second, the dynamics of choriocapillaris and retinal pigment epithelium (RPE) interactions and blood-retina barrier (BRB) functions. Several promising studies have demonstrated the development of retinal neuronal organoids and MPS, from mouse stem cells (Eiraku et al., 2011) to human stem cells (Nakano et al., 2012), from generating photoreceptors (Zhong et al., 2014) to retinal ganglion cells (Ohlemacher et al., 2016). Retinal organoids have, to this point, been developed by isolating human photoreceptor precursors and self-assembling of layers of differentiated photoreceptors (Chen et al., 2016; Lakowski et al., 2018), or from human iPSCs (Zhong et al., 2014). Most of the retinal neuronal cells have limited capacity for regeneration where iPSCs cells have the capability to differentiate into functional retinal neuronal cells. By using retinal organoids, precursor cells, and their microenvironment, the retinal MPS models are emerging to recapitulate parts of retinal functions. A microfluidic chip to study synaptic regeneration has been developed (Su et al., 2015). In the study, retinal precursor cells were isolated and cultured in a microfluidic chip with multiple arrays of microchannels to restore the retinal neuronal synapse. Another study utilized multi-passage mouse retinal progenitor cells, leading to the development of a μRetina chip (Mishra et al., 2015). By coupling computer simulations and experimental validations, chemical concentration gradients are monitored with real-time imaging on cell migration.

Next, to emulate retinal vasculature/RPE interactions and BRB functions, MPS has the advantage of retaining the vasculature structure and co-culturing with RPE cells. An early model consisted of a simple co-culture chip with human RPE cell line ARPE-19 and human umbilical vein endothelial cells (HUVEC) (Kaji et al., 2014), and was followed up by a choroid chip with an artificial Bruch’s membrane (Chen et al., 2017). In recent times, an angiogenesis model is established with perfusable blood vessel networks, enabling the observation of pathological retinal angiogenesis (Chung et al., 2018). Another study has featured a BRB model using microfluidic system and evaluate the integrity of the epithelial and endothelial barrier function (Yeste et al., 2017). To date, new processes are emerging that seek to combine organoids and MPS to generate complex multi-layer retinal vasculature and RPE models (**Figure 1D**). One novel MPS, in particular, is being developed by integrating more than seven human iPSCs-derived retinal cell types (Achberger et al., 2019). This allows for the formation of outer segment-like structures and the establishment of *in vivo*-like physiological processes such as outer segment phagocytosis and calcium dynamics.

Remarkably, besides physiological models, some retinal organoid models have been shown to have an impact on investigating disease mechanisms. A 3D retina organoid has been established for studying X-linked retinitis pigmentosa (Megaw et al., 2017) and another organoid model has been recreated to study glaucoma using patient-derived samples (Ohlemacher et al., 2016). In addition, an AMD model has been employed to mimic chronic and acute mechanical stress on RPE cells during different stages of AMD (Farjood and Vargis, 2018). However, there is still only a limited number of retinal organoids and MPS models that are present.

### Cornea Models

Compared to the retina, the corneal structure is less complicated. There are critical design principles to generate functional corneal organoids. These principles involve: semitransparency, structural retaining corneal epithelium, stroma (organized collagen ﬁbrils), Bowman’s membrane/Descemet’s membrane, and endothelium (**Figure 1A**, left). Limbal stem cells/progenitor cells which reside in the peripheral of cornea, are the major source of corneal cell regenerations and tissue repair. iPSCs become an important alternative source of corneal cells. It is the first generation of corneal epithelial cells from iPSCs which are derived from dermal fibroblasts and the corneal limbal epithelium was reported in 2012 (Hayashi et al., 2012). A number of contemporary studies describe a novel method to generate three-dimensional corneal organoids from human iPSCs (Foster et al., 2017; Susaimanickam et al., 2017) (**Figure 1C**). These mini-corneal organoids, that ranging in size from one to seven millimeters in diameter, reproduce the early developmental events *in vitro* and duplicate similar anatomical features and gene expression profiles of corneas (Foster et al., 2017; Susaimanickam et al., 2017). However, more accurate differentiation protocol is needed in order to better mimic corneal functions and conditions like Fuch’s dystrophy or Keratoconus.

Besides organoids, corneal MPS are powerful models in studying corneal physiology and pathology. The first corneal-on a chip assay was in 2009, where the study developed a microfluidic device containing collagen vitrigel (CV) for the development of corneal microtissue patches (Puleo et al., 2009). Another example is an eye-on-a-chip that models blinking by integrating a hydrogel “eyelid” (Seo et al., 2019) to evaluate cornea therapeutic drugs and provided a realistic platform to prevent dry eye disease. The further development of these models would greatly benefit future pharmacological ocular drug topical delivery.

### Lens and Other Ocular Tissue Models

The advancement of novel retinal and corneal models has opened new ways for the development of other ocular tissue counterparts. Nonetheless, there are even fewer organoids and MPS models on lens and other ocular tissue, in comparison to corneal and retinal models. There is an organoid lens model for defining molecular disease mechanisms caused by cataract risk factors (Murphy et al., 2018). This study, in question, demonstrated the generation of light-focusing human micro-lenses from spheroidal masses of human lens epithelial cells purified from differentiating pluripotent stem cells. One further example involved a contact lens-on-a-chip system. The design facilitated the study of different disinfection agents to prevent severe eye infections (Guan et al., 2016). There are great potentials for these physiological and pathological models in accelerating the identification and screening of ophthalmic drug targets, to address such pathological conditions as cataract and glaucoma.

### Prospective and Future Directions

To date, there is still a lack of a perfect organoid or MPS model to capture the development process of an entire organ. Researchers are now seeking ways to develop more advanced models without sacrificing their reproducibility. Besides organoids and MPS applications in regenerative medicine (Sasai, 2013), another major pharmaceutical application is in drug screening, hit identifications, and lead optimizations. There are 3D high content drug screening models based on organoids and MPS (Aref et al., 2013; Bai et al., 2015b; Kim et al., 2015) that have been constituted for various other organs. The methodological concepts from the aforementioned studies could be applied to ophthalmic organoids and MPS models. It is imperative to generate robust ocular disease models to facilitate the evaluation of preclinical candidates, such as drug effects, toxicology, pharmacokinetics, and pharmacodynamics, for both synthetic drug candidates (Kondo and Inoue, 2019) and natural compounds (Bai et al., 2019; Sun and Zhang, 2019).

In addition to an integrative “organoid-on-a-chip”(Park et al., 2019), a future direction of ocular MPS design should focus on mirroring the development of eye tissue on-chip, with special tissue fidelity features, such as vascularized tissue, ECM, immune cell interactions. Possibly, integration of complex functions, such as light-sensing and circuit structure in the vision system, would allow for capturing electric signals from the eye for probing vision problems in a noninvasive manner and toward more precise and controllable models.

The development of new technologies sheds new light on the understanding of ophthalmic disease mechanisms with the use of organoids and MPS models. Patient-derived stem cell organoid models would benefit personalized medicine. CRISPR/Cas9 technology and single-cell sequencing on-chip would subsequently enable complementary assay on basic research and clinical trials, moving toward a revolution in the conventional drug development pipeline. Finally, refined protocols of organoids and advances on microfluidic technology, 3D printing, biomaterials would potentially lead to integrative tissue models to recapitulate the physiological hallmarks of an entire eye. Although challenges still exist, more opportunities have arisen to improve the basic understanding of the ocular diseases and drug development to prevent vision loss.

## Author Contributions

JB and CW designed the review paper structure and layout. JB and CW contributed to the preparation of the manuscript.

## Funding

This study was funded by the Science and Technology Development Fund, Macau SAR (The additional fund to State Key Laboratory of Quality Research in Chinese Medicine).

## Conflict of Interest

The authors declare that the research was conducted in the absence of any commercial or financial relationships that could be construed as a potential conflict of interest.
